# Lactate Modulates the Activity of Primary Cortical Neurons through a Receptor-Mediated Pathway

**DOI:** 10.1371/journal.pone.0071721

**Published:** 2013-08-12

**Authors:** Luigi Bozzo, Julien Puyal, Jean-Yves Chatton

**Affiliations:** 1 Department of Fundamental Neurosciences, University of Lausanne, Lausanne, Switzerland; 2 Cellular Imaging Facility, University of Lausanne, Lausanne, Switzerland; Centre national de la recherche scientifique, University of Bordeaux, France

## Abstract

Lactate is increasingly described as an energy substrate of the brain. Beside this still debated metabolic role, lactate may have other effects on brain cells. Here, we describe lactate as a neuromodulator, able to influence the activity of cortical neurons. Neuronal excitability of mouse primary neurons was monitored by calcium imaging. When applied in conjunction with glucose, lactate induced a decrease in the spontaneous calcium spiking frequency of neurons. The effect was reversible and concentration dependent (IC_50_ ∼4.2 mM). To test whether lactate effects are dependent on energy metabolism, we applied the closely related substrate pyruvate (5 mM) or switched to different glucose concentrations (0.5 or 10 mM). None of these conditions reproduced the effect of lactate. Recently, a G_i_ protein-coupled receptor for lactate called HCA1 has been introduced. To test if this receptor is implicated in the observed lactate sensitivity, we incubated cells with pertussis toxin (PTX) an inhibitor of G_i_-protein. PTX prevented the decrease of neuronal activity by L-lactate. Moreover 3,5-dyhydroxybenzoic acid, a specific agonist of the HCA1 receptor, mimicked the action of lactate. This study indicates that lactate operates a negative feedback on neuronal activity by a receptor-mediated mechanism, independent from its intracellular metabolism.

## Introduction

The regulation of adequate energy supply is of prime importance for normal brain function. For this reason, the brain is equipped with efficient systems to sense and regulate the concentration of key energy substrates both centrally and peripherally. Lactate, present in the low millimolar range in the human and rodent brains [1]–[3], is increasingly presented as an alternative energy substrate to glucose for brain cells [4], [5]. A few studies have documented that lactate can influence the excitability of selected neurons via different metabolic pathways. In glucose-sensing neurons of the ventromedial hypothalamic nucleus (VHN), lactate was found to stimulate the action potential firing frequency [6]. In the subfornical organ, center for the control of salt-intake behavior, the firing of GABAergic neurons is regulated by lactate [7]. Lactate and glucose can share common mechanisms for neuronal modulation. Both molecules lead to the production of mitochondrial ATP, which leads to the modulation of selected membrane conductances such as K_ATP_ channels in glucose-excited neurons [8], [9], or hyperpolarizing chloride channels in glucose-inhibited neurons [10]. Studies have also demonstrated that, in some neurons, lactate and glucose effects are dissociated, such as in VHN glucose-inhibited neurons, where they have opposite effects [6], or in orexin neurons, where only lactate influences the firing frequency [11]. It is therefore conceivable that energy substrate-sensing systems are able to discriminate between different substrates, or that glucose and lactate do not encompass identical functions.

Studies in glucose-excited [12] and glucose-inhibited neurons [13] have found that glucose sensitivity is not always mediated by intracellular variations of ATP. It has even been proposed that a membrane receptor for glucose underlies its effects in glucose-inhibited neurons [14].

These considerations brought up the question of whether neurons can also selectively sense or respond to lactate with a ATP-independent mechanism. This would confer additional roles on lactate such as that of a signaling molecule for the brain cells, as has been recently proposed [15]. In support of this hypothesis, a family of G-coupled receptors has been recently identified [16] and called hydroxycarboxylic acid receptor (HCA, formerly named GPR81). Among them, HCA1 is considered to be a sensor for lactate in peripheral organs such as the adipose tissue [17], [18]. Recently, this receptor has been demonstrated to be expressed also in the adult brain [19]. The potential involvement of lactate receptors influencing neuronal activity has been proposed in a very recent review [20].

To explore these aspects, we investigated the influence of lactate application on the spiking output of mouse primary cortical neurons using rapid calcium imaging. Our results show that lactate can modulate neuronal network activity through receptor-mediated mechanisms.

## Materials and Methods

### Ethics Statement

Every effort was made to minimize suffering and the number of animals used in all experiments. All experimental procedures were carried out in strict accordance with the recommendations of the Swiss Ordinance on Animal Experimentation and were specifically approved for this study by the Veterinary Affair Office of the Canton of Vaud, Switzerland (authorization number 1288.5).

### Cell Culture

Mouse cortical neurons in primary cultures were obtained from E17 GAD67 EGFP knock-in C57bl6 or wild type C57bl6 mouse embryos. After removing meninges, entire cortices were first incubated with 180 U/ml trypsin for 20 min at 37°C and then mechanically dissociated in Neurobasal (Invitrogen, Basel, Switzerland) culture medium plus 10% FCS by successive aspiration through sterile glass pipettes. The dissociated cells were filtered using a cell strainer with 40 µm nylon mesh and re-suspended in Neurobasal culture medium complemented with 2% B27 and 500 µM GlutaMAX supplement (Invitrogen). Cells were then plated at a density of 20,000 cells per cm^2^ on glass coverslips coated with poly-D-lysine and laminin (Invitrogen). Half of the culture medium was exchanged each 5–6 days and cells were used, at DIV 14–21.

### Live Microscopy

Experiments were carried out on the stage of an upright epifluorescence microscope (Nikon, Tokyo, Japan) using a 40 × 0.8 N.A. water-immersion objective lens (Nikon). Fluorescence excitation wavelengths were selected using a fast filter wheel (Sutter Instr., Novato, CA) and fluorescence was detected using an Evolve EMCCD camera (Photometrics, Tucson, AZ). Digital image acquisition as well as time series were computer-controlled using the software Metafluor (Universal Imaging, West Chester, PA, USA). Up to 8 individual neurons were simultaneously analyzed in the selected field of view.

### pH Measurements

Intracellular pH (pH_i_) was measured in single cells on glass coverslips after loading the cells with the pH sensitive fluorescent dye 2′,7′-bis(carboxyethyl)-5,6-carboxyfluorescein (BCECF-AM; Teflabs, Austin, TX) as described previously [21]. Cell loading was performed at room temperature for 10 min using 1 µM BCECF-AM in a HEPES-buffered balanced solution (see composition below). Fluorescence was sequentially excited at 440 and 490 nm and detected through a 535 nm (35 nm bandwidth) emission interference filter. Fluorescence excitation ratios (F490 nm/F440 nm) were computed for each image pixel and produced ratio images of cells that were proportional with pH_i_. In situ calibration was performed after each experiment using a nigericin technique as described before [21].

### Calcium Measurements

Intracellular calcium was measured using the indicator Fluo-4 AM 5 µM (Teflabs Austin, TX) loaded for 15 min at 37°C. Experiments were performed in CO_2_/bicarbonate-buffered solutions (see composition below). Fluorescence was excited at 490 nm and detected at >515 nm, with an acquisition rate of 10 Hz. Fluorescence intensity was measured in regions of interest delineating the neuronal soma using Metafluor. Subsequently calcium transient extraction was performed using Minianalysis 6.0.3 (Synaptosoft Inc). The software includes an algorithm for the detection of complex and multiple events giving the possibility to detect overlapping or closely occurring peaks. Briefly, to identify the occurrence of a spike, the detection algorithm uses a combination of parameters such as running baseline analysis, signal amplitude threshold, area under the curve of signal above threshold, and period to search a local maximum. In a subset of experiments, to distinguish principal (glutamatergic) neurons and GABAergic neurons, we used cultures obtained from GAD67 EGFP knock-in mouse. Because green fluorescent protein (GFP) expressed by GABAergic cells and the calcium fluorescent dye Fluo-4 AM have overlapping excitation and emission spectra, we elaborated a strategy to distinguish them. The microscope was equipped with a motorized XY moving stage (Sutter) driven by custom-made software that allowed us to rapidly switch between selected XY positions. Before cells loading, a series of images were recorded in different fields of view in the same culture and their coordinates were stored. Cells were subsequently loaded with the Fluo-4 AM. By superimposing images at same XY positions, we were able to distinguish GFP positive and negative cells loaded with Fluo-4.

### Electrophysiological Recordings

Patch-clamp recordings were made with borosilicate glass pipettes with a resistance of 5.5–8 MΩ. The pipette solution contained (in mM): K-gluconate 130, NaCl 5, Na-phosphocreatine 10, MgCl_2_ 1, EGTA 0.02, HEPES 10, Mg-ATP 2, and Na_3_-GTP 0.5, pH 7.3 (adjusted with KOH).Recordings were made with an Multiclamp 700B amplifier (Molecular Devices). Data were acquired with a Digidata 1440A (Molecular Devices), at 10 kHz sampling rate, controlled with Pclamp 10 software and analyzed with Clampfit software (Molecular Devices). Series resistance was less than 15 MΩ and no compensation was used. A period of 5 min was routinely allowed after establishment of the whole-cell configuration. Experiments were performed using an open perfusion chamber. Control extracellular solutions and solutions containing the tested drugs were gravity fed at 600 µl/min and at 35°C on the cultured cells.

### Solutions

CO_2_/bicarbonate-buffered experimental solutions contained (mM): NaCl 135, KCl 5.4, NaHCO_3_ 25, CaCl_2_ 1.3, MgSO_4_ 0.8, NaH_2_PO_4_ 0.78, glucose 5, bubbled with 5% CO_2_/95% air. The pH of CO_2_-equilibrated solutions was 7.4, and was not altered by added lactate up to 20 mM. Glucose 5 mM was maintained in all solutions (unless otherwise specified). HEPES-buffered solutions contained (mM): NaCl 160, KCl 5.4, HEPES 20, CaCl_2_ 1.3, MgSO_4_ 0.8, NaH_2_PO_4_ 0.78, glucose 5, pH 7,3. For dye loading, this saline solution was supplemented with 0.1% Pluronic F127 (Molecular Probes, Eugene; OR) and glucose was increased to 20 mM. pH calibration solutions contained (mM): NaCl 20, KCl 120, HEPES 10, CaCl_2_ 1.3, MgSO_4_ 0.8, and NaH_2_PO_4_ 0.78 and were adjusted to their respective pH by addition of NaOH.

Pertussis toxin was from Tocris Bioscience (Zurich). Unless otherwise stated, all other compounds were from Sigma-Aldrich (Buchs, Switzerland).

### Immunocytochemistry

Primary mouse cortical cultures grown on coverslips were fixed with 4% paraformaldehyde in phosphate-buffered solution (PBS) for 15 minutes on ice. Cells were pre-incubated in PBS containing 15% serum and 0.05% Triton X-100 and subsequently incubated overnight with the primary mouse anti-NeuN antibody (1∶200, Millipore, Temecula, CA, USA) and rabbit anti-Gpr81 (Gpr81 is also known as HCA1) (1∶100, GPR81-S-296, Sigma). Cells were washed in PBS and incubated with the appropriate secondary antibodies (Alexa Fluor 488-conjugated donkey anti-mouse IgG and Alexa Fluor 594-conjugated donkey anti-rabbit IgG (Invitrogen). Negative controls were performed in the absence of primary antibodies. Coverslips were mounted in Fluorsave mounting medium (Calbiochem) and analyzed using a Leica SP5 confocal microscope and a 63× PlanApochromat objective lens with fluorescence excitation at 488 nm and 543 nm.

### Western Blot

Western blot was performed as described previously [22]. Briefly, mouse cortical neurons in primary cultures at DIV 14 were harvested in lysis buffer (20 mmol/L HEPES, pH 7.4, 10 mM NaCl, 3 mM MgCl_2_, 2.5 mM EGTA, 0.1 mM dithiothreitol, 50 mM NaF, 1 mM Na_3_VO_4_, 1% Triton X-100), and a protease inhibitor cocktail (Roche, 11873580001). Lysates were sonicated and protein concentration was determined using a Bradford assay. Proteins (25 µg) were separated by SDS-PAGE on a 12% polyacrylamide gel, incubated with an anti-Grp81 (HCA1) primary antibody (1∶500, Sigma) and then with a polyclonal goat anti-rabbit IgG conjugated with IRDye 800 (LI-COR, 926-32210). Protein bands were visualized using the Odyssey Infrared Imaging System (LI-COR).

### Data Analysis

Data are means ± SEM and are represented as percentage of spiking activity or ΔpH change measured during the control condition. In all experiments paired Student's t-tests were performed to assess the statistical significance (*P<0.05) except in the pertussis toxin experiments, where groups were compared with a non-paired Student’s t-test. The half-maximum inhibitory concentration (IC_50_) of L- or D- lactate was determined by non-linear curve fitting using the Levenberg–Marquardt algorithm implemented in the Kaleidagraph software package (Synergy Software, Reading, PA, USA). The concentration-response analysis experiments were fitted using the following equation:
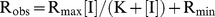
where R_obs_ is the observed response and R_max_, R_min_ are maximum and minimum parameters of the response. [I] is the concentration of the inhibitor compound and K is the concentration that yields its half-maximum inhibition (*i.e.* IC_50_).

## Results

### L-lactate Influences the Calcium Transient Frequency in the Presence of Glucose

**Figure 1:**
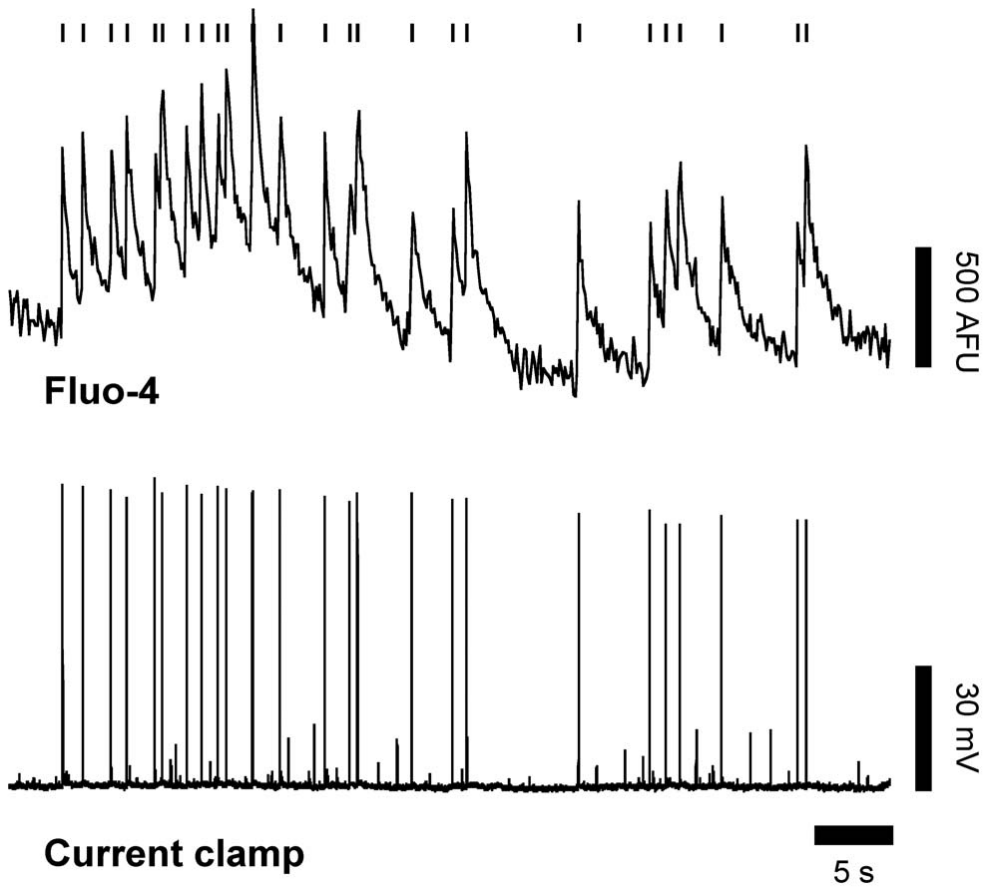
Neuronal activity monitored with calcium imaging. Comparison between simultaneous intracellular calcium imaging sampled at a frame rate of 10 Hz and whole-cell patch clamp recordings. A representative experiment out of 15 is shown with the upper trace representing calcium transients (arbitrary fluorescence units, AFU) and lower trace action potentials recorded in current-clamp configuration from the same neuron. The tick marks above the calcium trace indicate the occurrence of action potentials detected in the same cell using patch-clamp recordings.

The electrical activity of primary cortical neurons was monitored by calcium imaging. We took advantage of the fact that the membrane depolarization that accompanies action potentials leads to an intracellular increase of calcium concentration via the opening of voltage-gated calcium channels [23], [24]. During the time window of utilization of cells (DIV14-21), spontaneous calcium transients were detected in more than 50% of neurons. To assess to what extent the calcium transients correlate with action potentials in these cells, we performed simultaneous recordings in patch clamp and somatic calcium fluorescence. **Fig. 1** shows in parallel example traces of the spiking output recorded in whole-cell current clamp configuration and the corresponding intracellular variation of fluorescence that reflects the calcium variation. Careful visual inspection comparison of electrophysiological and optic recordings indicates an excellent match between both kinds of signals. The main advantages of the calcium imaging method for this study are that it allows monitoring a large number of cells in parallel and avoids altering the cellular solute composition. We therefore used calcium imaging in the following experiments as the main method for monitoring the electrical activity of neuronal population.

**Figure 2:**
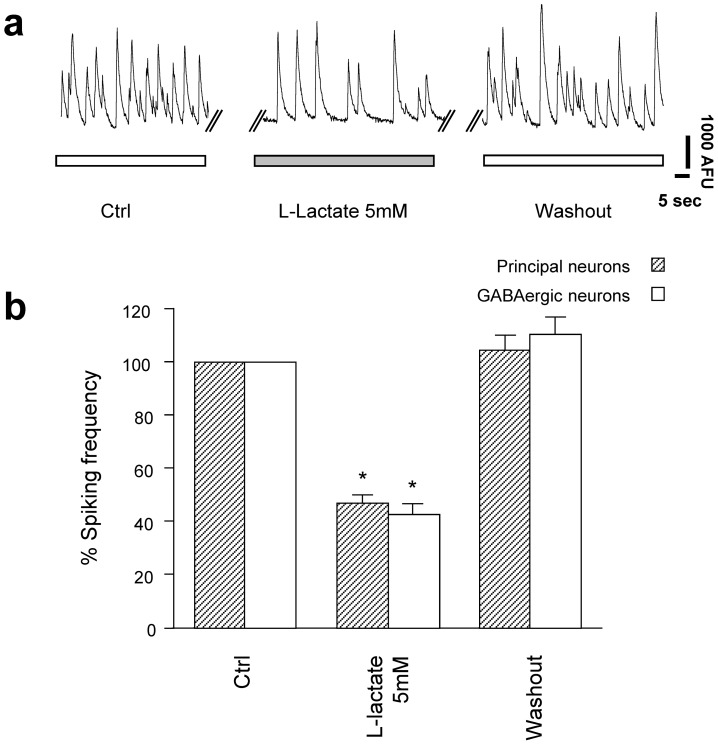
Effects of L-lactate on calcium spiking frequency. (a) Original traces of calcium transients in control or 5 mM L-lactate containing solution. (b) Calcium spiking frequency for principal glutamatergic neurons and GABAergic interneurons are shown as percent of activity measured during control solution. Data are obtained from 49 principal cells and 35 interneurons from 13 experiments.

**Figure 3:**
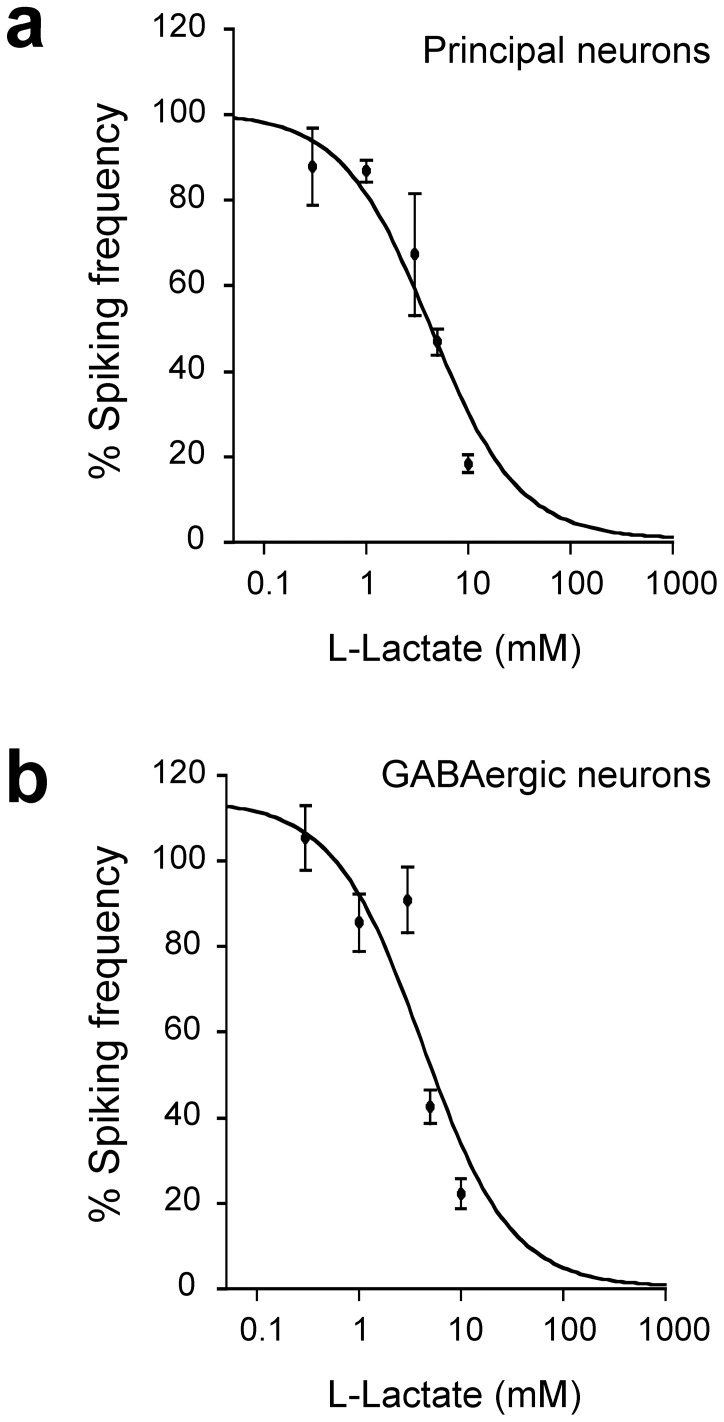
Concentration dependency of L-lactate effects. The decrease in calcium spiking frequency was concentration dependent. Apparent IC_50_ values obtained by nonlinear curve fitting yielded 4.2±1.9 mM for principal neurons (n = 175 cells, 56 exp) and 4.2±2.8 mM for GABAergic neurons (n = 83 cells, 35 exp).

A few studies have indicated that lactate could play a role as neuromodulator of certain glutamatergic and GABAergic neurons [6], [7], [12]. For this reason, we investigated the effect of L-lactate application on primary cortical neurons obtained from wild-type and GAD67 EGFP knock-in mice, which allowed us to distinguish principal glutamatergic neurons from GABAergic neurons. In order to investigate the modulatory effect of lactate and not its mere ability to sustain neuronal energy metabolism, experiments were carried out in the presence of 5 mM glucose. Recordings were obtained from the same target cells first in control solution, then following 5 min of L-lactate or other compounds application and ultimately after 5 min washout. **Fig. 2a** shows a typical experimental trace of the calcium transients in control or in the presence of L-lactate in a single cortical principal neuron. Application of L-lactate 5 mM reversibly diminished the calcium transient frequency by more than 50% in both principal and GABAergic neurons. **Fig. 2b** summarizes the results obtained in this series of experiments. In order to understand if the effect was proportional to the concentration, an inhibitory curve of L-lactate was then established **(** **Fig. 3** **)**. The graph shows that L-lactate decreased the calcium transient frequency in a concentration dependent manner in both cell types (apparent IC_50_: principal neurons 4.2±1.9 mM; GABAergic neurons 4.2±2.8 mM). As the sensitivity to lactate was found identical between principal and GABAergic neurons, cell types were not studied separately in the rest of the study. We also investigated whether the spontaneous calcium spiking frequency of individual neurons in the control period influenced the degree of inhibition caused by lactate. **Fig. S1** shows that the decrease in spiking frequency during lactate application was not correlated with the individual spiking frequency in the control condition.

### Related Energy Metabolites do not Influence Neuronal Activity

**Figure 4:**
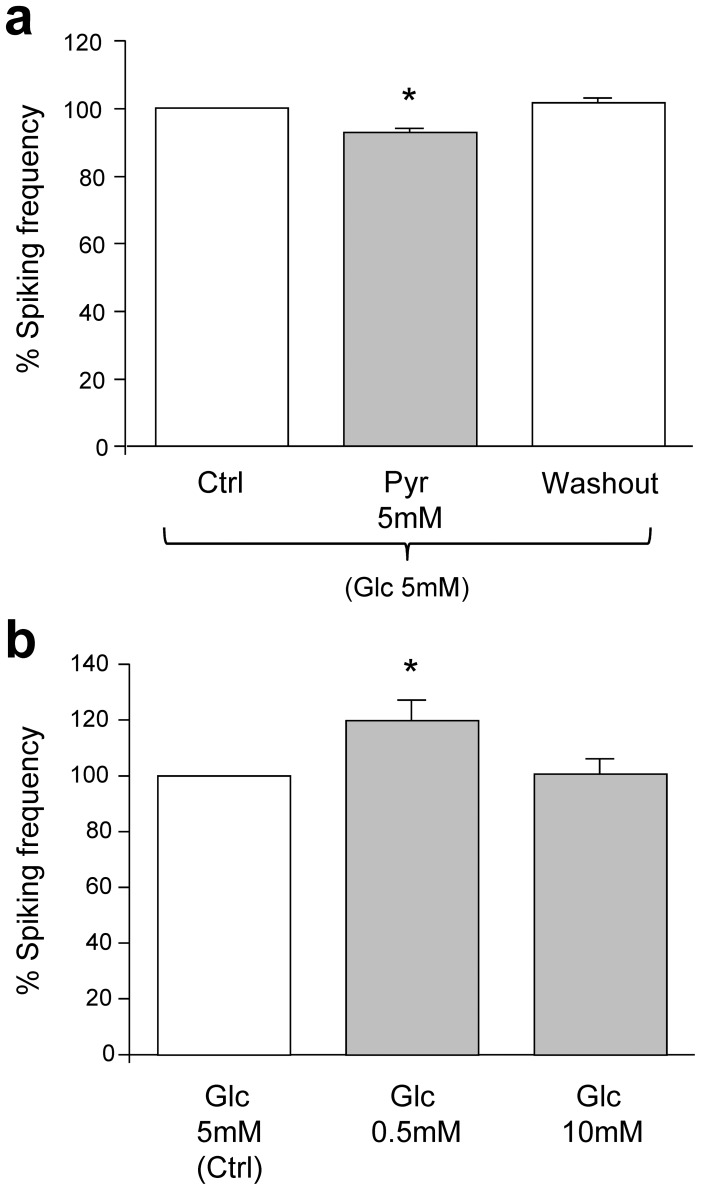
Energy metabolite dependency of calcium spiking frequency. Calcium spikes frequency shown as percent of activity measured during control solution. (a) Effects of pyruvate on calcium spiking frequency (n = 188 cells, 24 exp). Glucose (5 mM) was present throughout the experiments. (b) Effects of glucose concentration on spiking frequency (n = 68 cells, 10 exp).

In the cytosol of neurons, L-lactate can be converted into pyruvate by lactate dehydrogenase and then enter the tricarboxylic acid cycle that leads to the production of mitochondrial ATP. The ability of lactate to influence the neuronal activity could arise from the variation of intracellular ATP that influences directly or indirectly selected membrane conductances. We therefore examined whether related energy substrates cause a similar effect as L-lactate. We applied the same experimental protocol using the closely related molecule pyruvate or different concentrations of glucose (0.5 or 10 mM). Pyruvate 5 mM (in the presence of 5 mM glucose) marginally (∼7%) influenced the calcium transient frequency **(** **Fig. 4a** **)**. Glucose at high concentration (10 mM) did not replicate the effects of L-lactate, whereas low glucose (0.5 mM) tended to somewhat increase the frequency **(** **Fig. 4b** **)**. These experiments provided a first indication of the specific nature of lactate effects on neuronal activity.

### Effect of the Stereoisomer D-lactate

**Figure 5:**
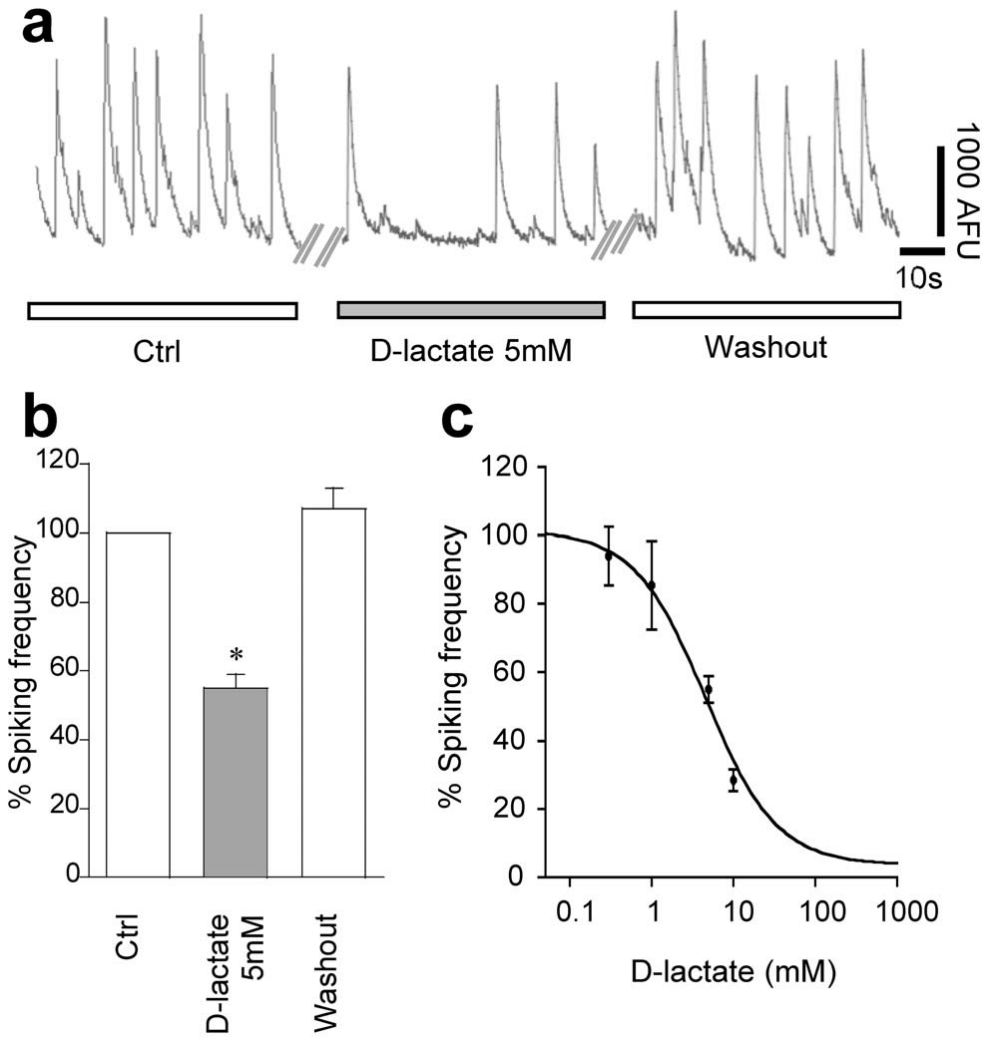
D-lactate effects on neuronal activity. (a) Sample trace of calcium transients in control or 5 mM D-lactate containing solution. (b) D-lactate substantially decreased calcium transient frequency. (c) The concentration-response analysis yielded an apparent IC_50_ of 4.6±1.2 mM (n = 127 cells; 21exp).

The above results suggest that in our experimental conditions intracellular production of energy equivalents is not involved in the observed modulation of neuronal activity. We further investigated the involvement of metabolism in the lactate effects by applying the stereoisomer D-lactate that is described to be poorly metabolized by neurons [25]. **Fig. 5a&b** show that D-lactate application substantially decreased the calcium transient frequency in a reversible manner. This effect was found to be concentration dependent with an IC_50_ of 4.6±1.2 mM **(** **Fig. 5c** **)**, *i.e.* approximately the same potency as the L-isomer (see **Fig. 3**).

**Figure 6:**
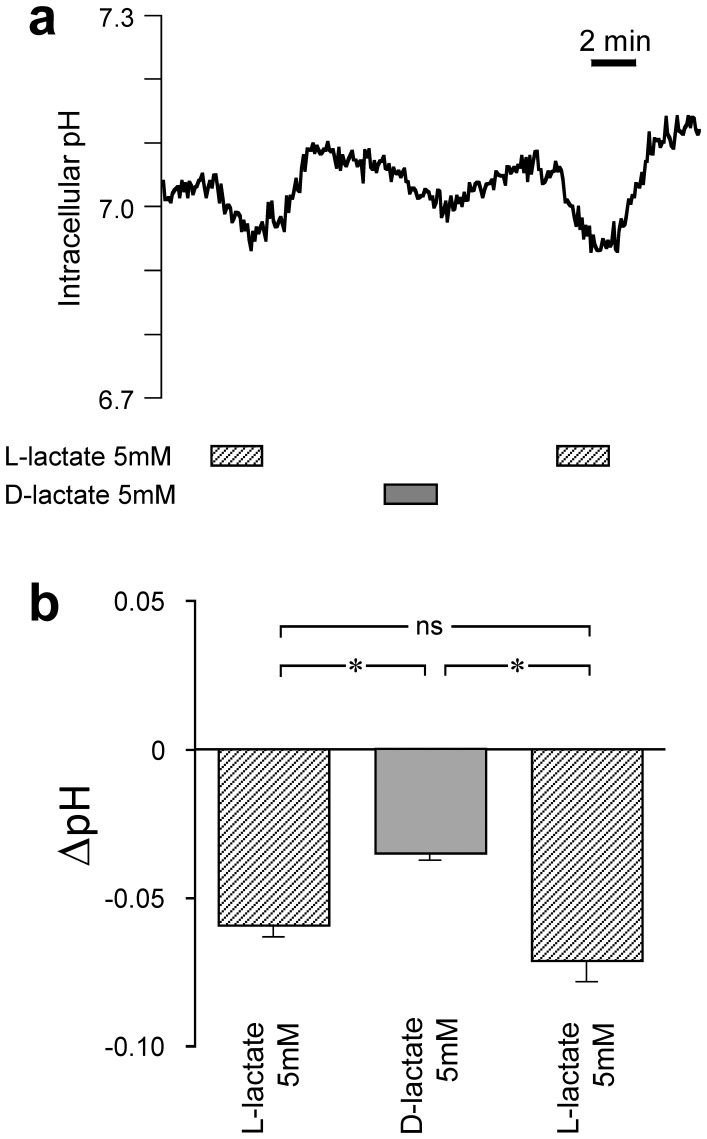
Intracellular pH effects of lactate isomers on cortical neurons. Intracellular pH measured using BCECF and calibrated *in situ* in cortical neurons. (a) Original pH trace during sequences of L- and D-lactate application. (b) Summary of acidification (pH amplitude) measured during L- and D-lactate application. (n = 39 cells; 7exp).

Both L-lactate and pyruvate are efficiently transported across the cell membrane of cortical neurons by monocarboxylate transporters (MCTs) [21]. It has been reported that neuronal MCTs transport D-lactate less efficiently [26]. To test to what extent, in our experimental conditions, D-lactate is transported into neurons, we took advantage of the fact that MCTs co-transport lactate with one proton with a stoichiometry of 1∶1 [21]. The resulting cellular acidification can be used to monitor the transport. The intracellular pH was monitored by loading neurons with the pH sensitive indicator BCECF and we used L-lactate application as control of the transport activity. **Fig. 6** shows that L-lactate application (5 mM) resulted in a small and reversible acidification (<−0.1 pH units). The figure shows that the acidification caused by D-lactate was significantly weaker than that caused by L-lactate at the same concentration, indicating that D-lactate is transported less efficiently into neurons than L-lactate as observed before [26]. These results strengthened the notions that lactate effects on spiking frequency are not solely related to its transport or intracellular metabolism.

### Receptor Mediated Effect of Lactate

**Figure 7:**
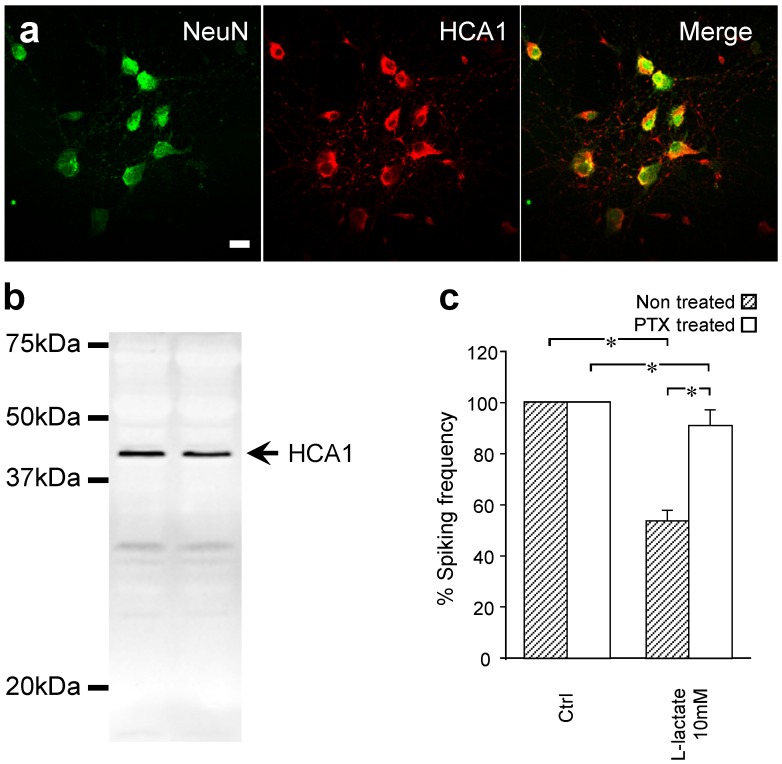
HCA1 receptor involvement in the lactate sensitivity. (a) Confocal images showing immunostaining for NeuN (green), HCA1 (red) and the merged image in mouse primary cortical neurons. Scale bar, 20 µm. (b) Representative Western blot showing that HCA1 is expressed in mouse primary cortical neuronal cultures. Each track represents one independent cultured dish of mouse primary cortical neurons (c) Comparison of lactate effect on calcium spiking frequency in cells incubated or not with pertussis toxin (PTX). PTX incubation strongly reduced the effects of lactate on neuronal activity. Data are obtained from 8 experiments and 61 cells for non-treated group and 8 experiments and 62 cells for PTX treated group.

Recently, a G-protein coupled family of receptors has been identified from a pool of orphan receptors and called hydroxycarboxylic acid receptor (HCA) [16]. Among them HCA1 (previously known as GPR81) was reported to be activated by lactate in adipocytes [17] and the brain [19]. To determine if this receptor is expressed by mouse primary cortical neurons, we performed an immunohistochemistry analysis using anti-HCA1 antibody. We found that all cells positive for the neuronal marker NeuN show HCA1 immunoreactivity in our primary cortical cultures (**Fig. 7a**). We also verified the antibody specificity and confirm that HCA1 is expressed in mouse cortical neuronal cultures by Western blot (**Fig. 7b**), that displays a band corresponding to the expected 40 kD molecular mass of the protein [27].

HCA receptors are reported to be coupled to G_i_ proteins [18]. To investigate whether a G_i_ coupled receptor is implicated in the observed lactate sensitivity we incubated cells with pertussis toxin (PTX), a G_i_ protein inactivator. Neuronal cultures coming from the same preparation were divided into two equal groups, one used as control and the other incubated with PTX (500 ng/ml, 24 h). Experiments were performed in parallel on the same day. Importantly, incubation of neurons with PTX did not significantly influence their basal spontaneous spiking frequency: non-treated neurons: 6.41±0.44 spikes/min (61 cells, 8 experiments), PTX treated neurons: 5.35±0.47 spikes/min (n = 62 cells, 8 experiments), p>0.05. In control condition, L-lactate induced the previously observed reduction of the calcium transient frequency by 46%. However, in the presence of PTX, the inhibitory effects of lactate were almost abolished (90.8±6% of the initial frequency was maintained, **Fig. 7c**).

**Figure 8:**
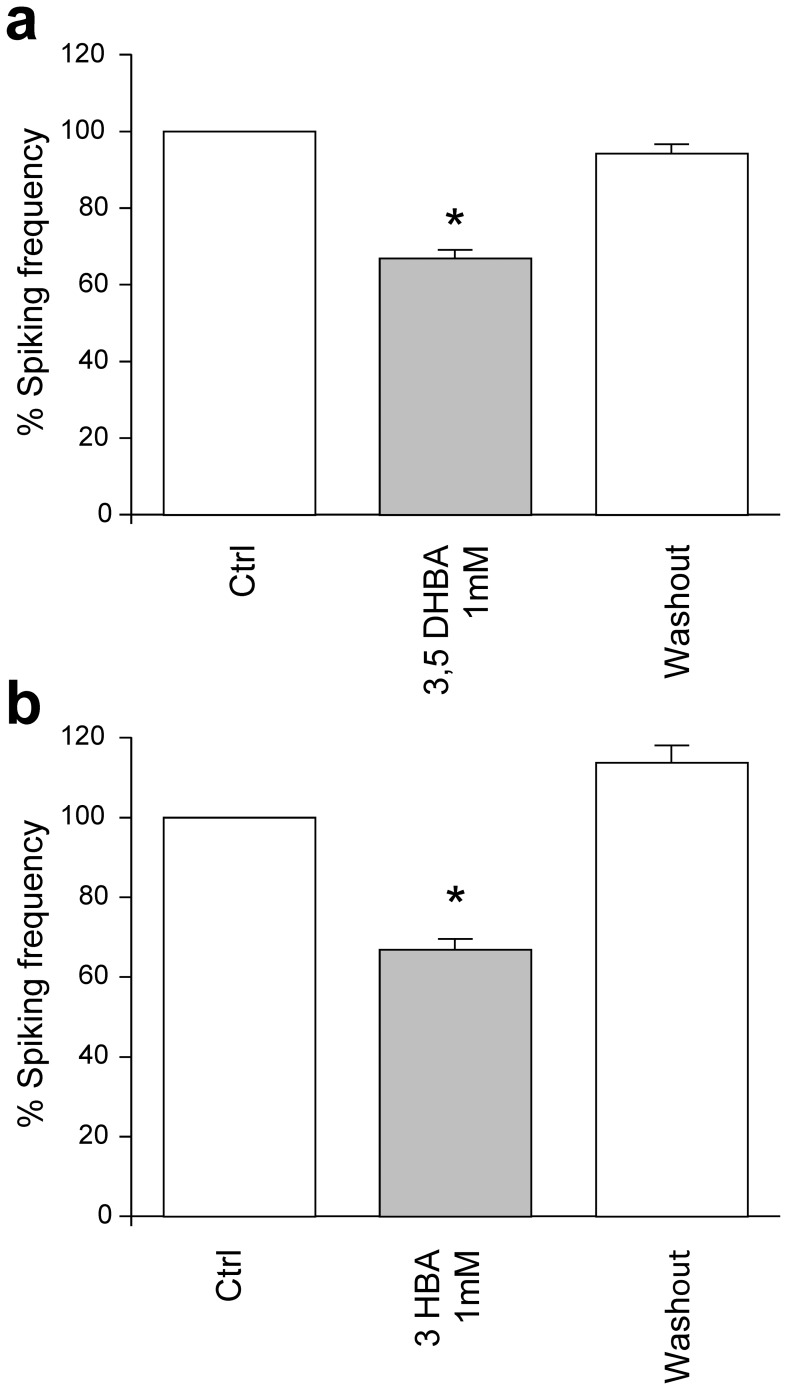
Reduction by 3,5-DHBA and 3-HBA of the calcium spiking frequency. Calcium spiking frequency shown as percent of activity measured during control solution. (a) Effects of 3,5-DHBA on calcium spiking frequency (n = 155 from 22 experiments). (b) Effects of 3-HBA on spiking frequency (n = 10 from 79 experiments).

To further investigate the involvement of HCA receptors, we tested the effects of 3,5-dyhydroxybenzoic acid (3,5-DHBA) recently identified as a specific agonist of the lactate receptor HCA1 [28] as well as 3-hydroxybenzoic acid (3-HBA) an agonist of HCA1 and HCA2, a receptor highly homologous to HCA1. Both agonists have been reported to have a higher affinity than lactate for these receptors, and were applied at a concentration of 1 mM. **Fig. 8** shows that, like L-lactate, both 3,5-DHBA (a) and 3-HBA (b) decreased in a reversible manner the neuronal activity by ∼33%.

## Discussion

This study shows for the first time that lactate administration to cortical neurons induces a distinct modulation in a way and magnitude that was not shared by other energy substrates. The observed modulation is driven by a mechanism likely involving a membrane receptor for lactate and independent of its metabolism.

We found that L-lactate application decreased the network activity of neurons in a concentration dependent manner in the presence of glucose. Interestingly, modulation of neuronal activity by lactate has been found in GABAergic neurons of the subfornical organ [7]. L-lactate in the concentration range 0–1 mM promoted the firing rate whereas in the range 1–10 mM the firing rate was progressively suppressed. In that study, the authors highlighted the stimulatory phase that they found to be ATP dependent; however, they did not attempt to explain the suppression of activity by higher lactate concentration, leaving the question open.

Among the different energy substrates used in our study, L-lactate was the only one able to strongly reduce neuronal firing frequency. High levels of glucose, or pyruvate applied at the same concentration as L-lactate, did not reproduce the effect. It should be noted that pyruvate is not only closely related to lactate but also transported by MCTs. While the MTC2 isoform (expressed in oocytes) was reported to have a lower Km for pyruvate than for lactate [29], [30], pyruvate was found to be very efficiently transported in mouse cortical neurons [21]. The ability of L-lactate to selectively influence neuronal activity was already found in orexin neurons where L-lactate–but not glucose–increased the firing activity of neurons [11]. The effects of energy substrates are usually assumed to be mediated by the intracellular variation of ATP. However, the disparity in the effect of these energy substrates led us to consider the possibility that the mechanism involved in the L-lactate sensitivity is not dependent on the levels of ATP produced. A growing body of evidence indicates that mechanisms underlying energy substrate sensitivity are not all dependent on the intracellular ATP concentration changes [12], [31]. We found that D-lactate, the stereoisomer of lactate that is poorly metabolized by neurons [32], induced the same effect as L-lactate with a very similar IC_50_. These results suggest that the mechanism of L-lactate sensitivity does not involve cellular energy metabolism.

An interesting parallel can be drawn with a study performed on glucose-inhibited neurons in the lateral hypothalamus [13], where only glucose–but not lactate or pyruvate–suppressed the firing activity. Moreover, the non-metabolized glucose analogue 2-deoxyglucose mimicked the effect of glucose, indicating that the glucose-induced hyperpolarization does not require glucose metabolism. In a previous study of the same group on the mechanisms involved in glucose-inhibited neurons [14], it was shown that glucose induced a K^+^ hyperpolarizing current that was caused only by its extracellular and not intracellular application. To explain this puzzling observation, the authors proposed the involvement of an extracellular glucose receptor.

An alternative mechanism that should be considered for the effects of lactate is intracellular acidification. Lactate is taken up by neuronal MCTs, which co-transport one proton together with lactate, and therefore can bring about cytosolic acidification. In our experiments, the addition of L- or D-lactate (5 mM) caused only a minimal acidification of 0.05–0.1 pH units. These pH changes are in agreement with published values of acidification by L-lactate [21] and by D-lactate [26] and appear unlikely to affect the spiking activity. The fact that D-lactate decreased neuronal activity with the same extent and potency than L-lactate but with a two-fold lower intracellular acidification indicates that the observed effect is not proportional to the pH variation. In addition, pyruvate was reported in a previous study on the same cells [21], to induce a larger intracellular acidification than L-lactate, whereas on the contrary we found it not to influence neuronal network activity as L-lactate.

Besides being poorly metabolized, D-lactate is less internalized in neurons than L-lactate, which was demonstrated by the lower acidification induced by the monocarboxylate transporter activation. This is consistent with the reported lower affinity of MCTs for D-lactate compared to L-lactate [26], [33]. Inasmuch as both isomers reduced the frequency of spiking to the same extent and with the same potency, it is plausible that L-and D-lactate do not need to enter neurons to induce their effects nor rely on MCT activity, and therefore act as an extracellular ligand.

A new class of G_i_ protein-coupled receptors has been recently, identified [16] with affinity for several intermediates of energy metabolism. The ligands being all hydroxyl-carboxylic acids (HCA), these receptors have been named HCA receptors. Of particular interest for the present study, the HCA1 isoform (previously known as GPR81) is described as a receptor for lactate with half-maximal affinity of 4.8 mM [18], very close to our measured IC_50_ value of 4.2 mM. The receptor is predominantly expressed in adipose tissue [18], but we found that it is also expressed in primary cortical neurons. This finding is in agreement with a recent paper that shows HCA1 localization in neurons in different regions of the brain such as cortex, hippocampus and cerebellum [19].

To test if such a receptor is implicated in the lactate sensitivity of neurons, we used 3,5-DHBA a specific agonist for the receptor recently discovered [28]. We found that the application of this agonist decreased the calcium transient frequency by 33% at a concentration of 1 mM. In addition, the application of 3-HBA an agonist that has a similar affinity for HCA1 but in addition can also bind to HCA2 receptor, highly homologous to HCA1, produced the same intensity of effect at equal concentration. These compounds induced a stronger inhibition of activity than lactate at the same concentration, which is consistent with their reported higher affinity for the receptor. Also, these receptors do not bind pyruvate, consistent with our observed lack of effect of pyruvate on spiking activity [17], [18]. However, literature shows discrepancies regarding the affinity of HCA1 for D-lactate, which we find, in cortical neurons, to have similar potency as L-lactate [17], [18]. Another indication of HCA1 involvement, in absence of a specific inhibitor currently available, came from PTX experiments. This inhibitor of G_i_ proteins almost abolished the decrease of neuronal network activity caused by L-lactate without altering the basal rate of spontaneous spiking activity. Taken together, this body of evidence strongly points to the involvement of HCA receptors in the described lactate sensitivity of neurons. Lactate binding to these G_i_-coupled receptors reduces the formation of cAMP via inhibition of the adenylate cyclase. A possible downstream effect of decreased cAMP is the reduction of exocytosis via a protein kinase A dependent pathway [34]. Another possible effector for the inhibition is based on the activation of the associated G_βα_ subunits that could induce an hyperpolarization by the opening of K^+^ conductances or reduce the exocytosis as was reported for the activation of GABA_B_ receptor, another G_i_ protein-coupled receptor [35].

The inhibitory activity of lactate could play several roles in the regulation of neuronal activity, and act as a paracrine element that prevents an excess of activity of neurons. Brain lactate level is estimated to be in the low millimolar range [1]–[3] and its concentration approximately doubles during neuronal activation [36]. While astrocytes are often described as the main lactate producers of the brain, lactate can originate from other sources. It has been proposed that other cell types than astrocytes in the brain, including neurons, could release lactate [37] and that, during intense physical exercise, increased peripheral lactate could enter the brain and be used [38]. In case of excessive neuronal activity, *e.g.* as it occurs during epileptic seizures, the increased levels of lactate may have the beneficial effects of calming down the network. In support of this hypothesis, it has been demonstrated that L-lactate reduced the size of lesion induced by glutamate in rat cortex [39]. Lactate application has been investigated in several brain disturbances and induced variable degrees of benefit, *e.g.* in cerebral ischemia [40]–[42], hypoxia [43], [44], traumatic brain injury [45] and hypoglycemia [46]. It is plausible that the new neuromodulatory role of lactate described in the present study could underlie some of its positive effects.

In conclusion, the results of this study allow us to propose a new role of lactate as a cellular signaling element providing a metabolic feedback for the modulation of neuronal activity.

## Supporting Information

Figure S1 **Lack of relationship between the individual neuron basal spiking frequency and its reduction by lactate.** (a) Spontaneous calcium spiking frequency (min^−1^) is depicted for individual neurons during the control condition, during 5 mM lactate superfusion, and during recovery period after washout of lactate. Data were obtained from 84 cells (13 experiments). (b) Change in calcium spiking frequency during lactate superfusion (shown as percent of the frequency in the control period) plotted against basal spontaneous calcium spiking frequency (min^−1^) observed during the control period for each individual cell (data collected from panel a).
(TIF)
