# Supplementary figures and images for: Lactate Modulates the Activity of Primary Cortical Neurons through a Receptor-Mediated Pathway
Source: PLoS One. 2013 Aug 12;8(8):e71721. doi: 10.1371/journal.pone.0071721 (PMC3741165; doi:10.1371/journal.pone.0071721)

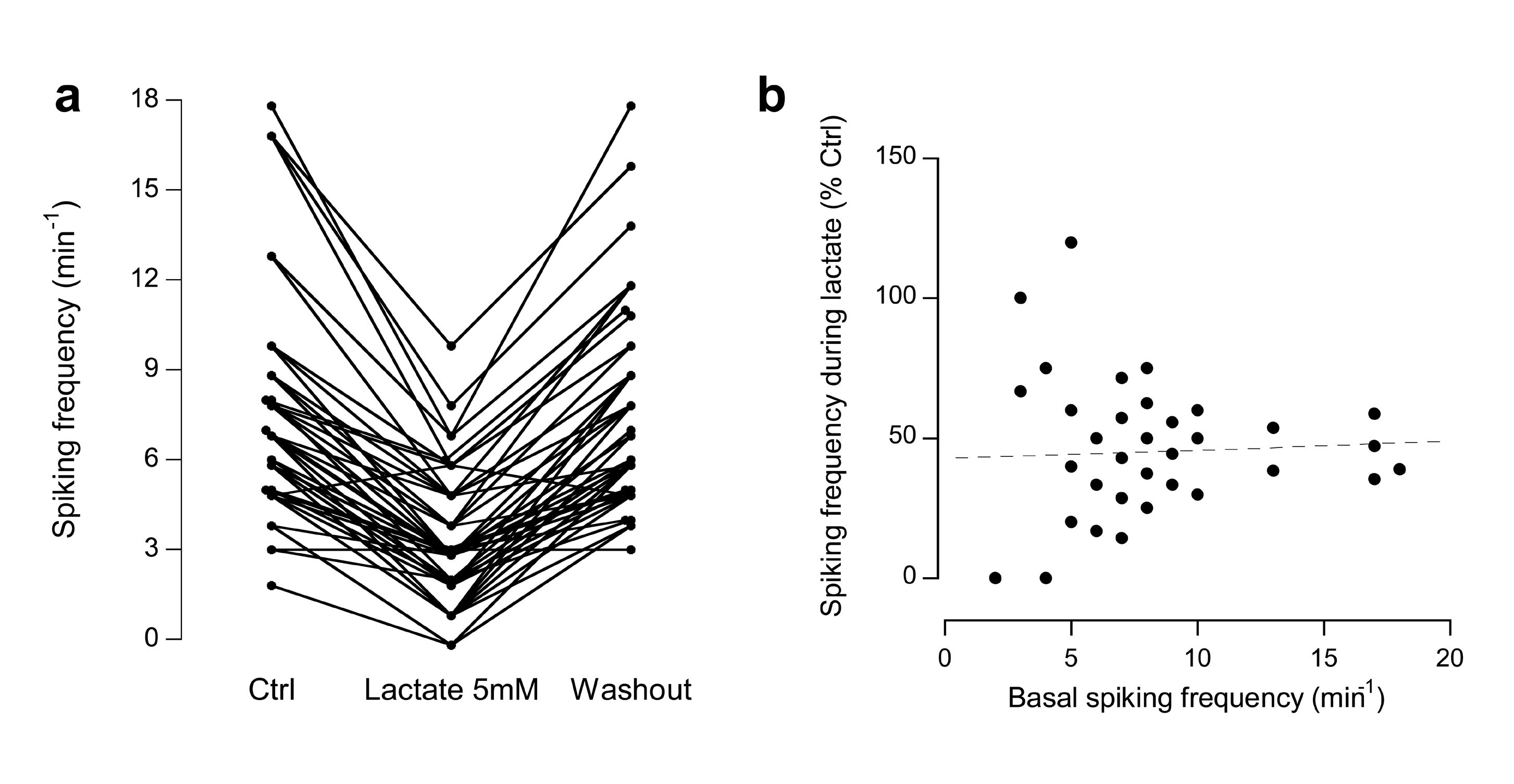

Supplement: Figure S1 — Lack of relationship between the individual neuron basal spiking frequency and its reduction by lactate. (a) Spontaneous calcium spiking frequency (min−1) is depicted for individual neurons during the control condition, during 5 mM lactate superfusion, and during recovery period after washout of lactate. Data were obtained from 84 cells (13 experiments). (b) Change in calcium spiking frequency during lactate superfusion (shown as percent of the frequency in the control period) plotted against basal spontaneous calcium spiking frequency (min−1) observed during the control period for each individual cell (data collected from panel a). (TIF) [file pone.0071721.s001.tif]
